# Access to Interpretable Data to Support Disproportionate Health Risks from Industrial Releases: A Case Study on the Environmental Protection Agency’s Datasets and Their Application to the Latinx Communities of Houston, Texas

**DOI:** 10.3390/ijerph22020291

**Published:** 2025-02-16

**Authors:** Hannah Wheless, Lori A. Hoepner

**Affiliations:** 1 Gallatin School of Individualized Study, New York University, New York, NY 10003, USA; 2 Department of Environmental and Occupational Health Sciences, School of Public Health, SUNY Downstate Health Sciences University, Brooklyn, NY 11203, USA; lori.hoepner@downstate.edu

**Keywords:** environmental justice, EPA, GIS, industrial emissions, Latinx health, RSEI, TRI

## Abstract

Latinx communities face disproportionate environmental injustices and are targeted due to systematic economic and political inequities. This research evaluates the ease at which links between industrial releases and risk of adverse health effects can be defined to influence policy change in Houston, TX. The Environmental Protection Agency (EPA)’s Toxic Release Inventory (TRI) is the most comprehensive public database on industrial facilities’ toxic chemical releases in the US. TRI is presented within a risk-based context through the Risk Screening Environmental Indicators (RSEI) scores. TRI and RSEI datasets for Houston in 2022 were assessed in QGIS to analyze chemical release and risk in neighborhoods using Community Tabulation Areas (CTAs), identifying demographics of communities facing disproportionate industrial releases and consequent potential health risks. Geospatial visualizations reflected Latinx communities to house the heaviest polluting industrial facilities in Houston. As a result, these communities face the highest potential risk of adverse health effects due to exposure to a multitude of chemicals—particularly 1,3-butadiene, benzene, and chromium—as reflected in cumulative RSEI scores. An analysis of TRI and RSEI datasets elucidates the burden of gathering and analyzing chemical release data in a public health context, reflecting why change beginning at the local level can be difficult for under-resourced Latinx communities facing industrial pollution. Improving the accessibility and utility of the EPA resources will provide a resource to advocate for data-driven policy change.

## 1. Introduction

The EPA defines Environmental Justice as “the fair treatment and meaningful involvement of all people regardless of race, color, national origin, or income with respect to the development, implementation, and enforcement of environmental laws, regulations, and policies” [1]. “Fair treatment” is then further explained by the EPA to mean that “no group of people should bear a disproportionate share of the negative environmental consequences resulting from industrial, governmental, and commercial operations or policies” [1].

BIPOC, working-class, and lower-income communities disproportionately experience environmental health injustices, with Latinx * populations in particular facing heightened health risks due to industrial pollution [2,3,4,5]. These injustices frequently come about in one of two ways, although they are not mutually exclusive over time: (1) companies developing strongly polluting facilities identify and move into neighborhoods with a high percentage of historically underrepresented residents because these communities are more likely to have less empowerment, lower education, and fewer financial resources, resulting in systematic economic and political barriers in combatting injustices [6,7]; and (2) the presence of toxic industrial facilities depresses land values, causing those with the privilege of mobility to flee and minority residents who have fewer housing options to move in as industrial neighborhoods are more affordable [8].

Houston, Texas, exemplifies environmental and public health injustices largely due to its status as one of the world’s largest ports and a city characterized by “unrestrained capitalism, unrestrained growth, and…industrial dependency upon petrochemical [industries]”, as described by Robert Bullard, the “father of environmental justice” [9,10]. The city is a major pollution hub, not just for being home to the largest petrochemical complex in the country and over 400 chemical manufacturing facilities, but also due to tailpipe emissions from residents driving more than 140 million miles daily [10]. As a result, while industrial and manufacturing facilities continue to thrive, Latinx communities face disproportionately high exposure to chemical releases, living in a “toxic normal”, where seeing or smelling hazardous chemicals in daily life is routine [11].

Latinx grassroots organizations in Houston have successfully built power at the local and state levels, influencing federal representatives and policies to enforce industrial practices that do not threaten the health of surrounding neighborhoods [7]. For instance, the Mexican American-run environmental justice organization t.e.j.a.s, (Texas Environmental Justice Advocacy Services) in the local Houston area, has seen significant victories through increased academic research on toxic chemicals and in-depth focuses on public health [11]. The prioritization of data-driven decision making through research is especially important in the effort to pass environmental justice policies, which are often premised on geographical analyses of demographic criteria and issues of environmental injustice [1]. The EPA has online resources pertaining to industrial facility releases and their modeled health risks that are available for public use. However, these resources and the software required to analyze the data may not be attainable or utilizable enough to truly be helpful for Latinx communities in advocating for improved living conditions.

The reality of the inaccessibility of these public datasets and resources reflects why less-resourced groups, more likely to face environmental injustices, are less likely to be able to utilize public EPA resources or software to provide scientifically supported proof of public health risks. By highlighting the steps and resources required to conduct a geospatial analysis on community demographics, industrial emissions, and related public health threats, this case study examines why communities disproportionately affected by environmental injustices may have difficulty providing scientific proof to advocate for change.

This paper questions which communities in Harris County face the greatest air emissions via industrial facilities, and if there is a specific demographic disproportionately affected. Additionally, to investigate any potential health effects from such levels of releases, this analysis examines if there are increased levels of chemicals that pose environmental health threats in these neighborhoods. To delve into such spatially related questions, this paper uses the Geographic Information System (GIS) software QGIS. Because GIS is an extremely powerful mapping and analysis technology that can analyze large quantities of data in a geographic context, it can provide solutions to spatial issues such as public health threats [12]. In turn, GIS has served as an accessible, reliable way to integrate mapping processes with health data to study spatial and temporal trends with risk [13]. Therefore, this software has been largely used to examine the spatial realities of environmental injustice issues by analyzing the geographic relationships between sources of pollution burdens, such as industrial facilities, and the characteristics of the populations at risk of being affected by such pollution [12]. For these reasons, QGIS 3.36.2-Maidenhead, a free and open-source GIS platform, was used to examine industrial facility pollution and potential health risks within the spatial context of the communities of Houston.

* This analysis used Census ethnicity data in which individuals were required to check either a “Hispanic” or “non-Hispanic” box. Although this paper is applicable to all Latinx individuals, the word “Hispanic” will be used in accordance with the data included in the analysis.

## 2. Materials and Methods

### 2.1. Study Area

Harris County serves as an ideal area for assessment as it is home to 360 facilities subject to TRI reporting and it has identifiable census blocks for evaluation of areas experiencing environmental injustice [14]. The county occupies 1707.0 square miles of land area in the upper Gulf Coast of Southeast Texas [15]. Additionally, the county contains 144 Census-defined areas, 73 of which report a majority Hispanic population [15]. As of 2022, Harris County’s population reached 4,726,177 people, with Hispanic individuals representing the largest ethnic group as 44% of the city’s population [15].

To break down Harris County into representative communities, Community Tabulation Areas (CTAs) were used. The Kinder Institute for Urban Research designed CTAs to divide Harris County into communities that would serve as better approximations of neighborhoods than simply zip codes, known as zip code tabulation areas (ZCTAs), which were used previously to define communities [16]. A CTA is a combination of one or more Census tracts dependent on factors like social community boundaries (deemed “super neighborhoods”), commercial areas, and school districts to form the most representative divisions of the neighborhoods in Harris County [16].

To also map demographic information such as race and ethnicity to the CTAs/neighborhoods of Houston, data from the Census’ American Community Survey (ACS) Five Year Estimate was used. Created by the United States Census Bureau, the ACS is an annual survey that provides information about the country and its citizens [15]. Through the ACS, the government takes information on ethnicity, race, occupation status, education attainment, housing status, and other topics to better plan how federal funds are distributed each year [15]. These data are used by researchers, public officials, planners, and entrepreneurs to examine the past and plan the future.

### 2.2. Step One: Analyzing Harris County for the Spatial Distribution of Industrial Pollution Burden

If a concerned civilian or researcher is interested in industrial facility emissions, the EPA’s Toxic Release Inventory (TRI) is ideal. Deemed the most comprehensive national public database on toxic chemicals in the U.S., the EPA’s TRI database, implemented in 1986, tracks industrial facilities’ management of chemicals with the potential to harm human health and the environment [14,17]. Thresholds exist for manufacturing, processing, and other use of a TRI chemical (25,000 lbs, 25,000 lbs, and 10,000 lbs, respectively). If an industrial facility reaches the threshold of a TRI chemical, they are then required to report their subsequent chemical releases annually [18]. Facilities that meet the criteria for TRI data collection must publicly disclose the amount (in pounds) of chemical releases on-site via air (stack and fugitive emissions), water, land, and underground injection, and transfers off-site for recycling or disposal, for 794 chemicals as of 2023 [18]. The most Hazardous Air Pollutants (HAPs) are TRI-reportable chemicals, but TRI includes some additional chemicals that are not classified as air toxics under the Clean Air Act [14]. To “help make the reported data more understandable”, the EPA provides multiple online tools with the TRI datasets, such as the TRI Toxics Tracker at https://edap.epa.gov/public/extensions/TRIToxicsTracker_embedded/TRIToxicsTracker_embedded.html?#home (accessed 12 February 2025). On this website, the public can search for the summaries of TRI facilities by state, county, zip code, and by an address, in which one would obtain results for within 10 miles of that address [14]. Through the facilities summary, the interface presents the breakdown of releases, waste management, pollution prevention, potential harm, and chemicals [14].

Data for environmentally released industrial chemicals in Harris County, Texas, in 2022 were obtained as spreadsheets from TRI, available for download on the EPA’s TRI website at www.epa.gov/tri/ (accessed 12 February 2025) [14]. In the Basic Data Files subsection of the TRI Data and Tools section, datasets are available and can be isolated by year and state containing (1) industrial facility name, address, and latitude and longitude coordinates, (2) chemical identification and classification information, (3) quantities of chemicals released on-site at the facility (separated into fugitive air, stack air, and water releases), (4) quantities of chemicals transferred to public treatment facilities, and (5) quantities of chemicals transferred off site to other locations for release/disposal/waste management [14]. The downloaded spreadsheet has rows for each chemical released by each industrial facility; meaning that there will be multiple rows for each facility—this may not be apparent at first glance as chemical releases are not grouped within the sheet. The data were then sorted alphabetically to better visualize the chemical release breakdown by industrial facility. For the purposes of this analysis, the spreadsheet included facility data for all of Texas in 2022 and was filtered by county selecting for only Harris County.

To analyze TRI data in a public health context, it is most useful to quantify the amount of air pollution released from industrial facilities, as one cannot deduce the scope of areas water releases will impact, or the areas that off-site releases would affect [11]. Since both fugitive and stack air releases often result in undetectable potential human exposure to airborne chemicals, these two counts of releases in annual pounds are of interest in an environmental health context. As pounds of chemicals released are separated into two distinct values for fugitive and stack, these two columns were combined into one summative air pollution column.

To visualize which communities of Houston are collectively more impacted by industrial air pollution, this analysis maps TRI air releases as summaries of Harris County’s CTAs. It is first necessary to download the shapefile of the geographical area of interest; in Houston’s case, the dataset catalog from the Rice University Kinder Institute for Urban Research’s Urban Data Platform has the shapefile defining the boundaries of Harris County under the Community Tabulation Areas 2020 dataset [16]. Next, the CTA shapefile was added as a layer in a project in QGIS, exported, and saved. The same was carried out for the TRI spreadsheet but with the layer properties’ symbology of the data represented with a graduated point, where the larger the point, the higher the pounds of chemicals released into the air. Then, the following steps were taken: join attributes by location (summary) and join to features in the CTA layer where the features intersect by comparing to the TRI air release data, with sum being the summary to calculate. After adjusting the symbology of the resulting joined later to be graduated by the sum of air release values, geospatial representations of the collective burden of industrial air pollution in Harris County were produced.

### 2.3. Step Two: Evaluating Individual Toxic Releases for the Spatial Distribution of Industrially Related Public Health Risk

As mentioned, TRI data are less useful for analyzing public health threats as this information is reported in pounds per year. Since the TRI pounds/year values cannot be compared to Minimal Risk Levels in milligrams/cubic meter to determine unsafe levels of air pollution, the EPA developed the Risk Screening Environmental Indicators (RSEI). RSEI is a model that incorporates TRI information to evaluate potential adverse health effects of industrial emissions from a risk-based perspective [19]. RSEI considers the amount and circumstances of the chemicals used, managed, or released into the environment, along with the toxic potential and likelihood of exposure [19]. Chemical toxicity and modeled risk scores are determined using multiple sources such as the EPA’s Integrated Risk Information System (IRIS), Air Toxics Screening Assessment (AirToxScreen), Office of Pesticide Programs (OPP), Provisional Peer-Reviewed Toxicity Values (PPRTVs), Health Effects Assessment Summary Tables (HEAST), along with the ATSDR and the California Environmental Protectional Agency (CalEPA) to determine a chemical’s toxicity and therefore its modeled risk scores [19]. This model also has a well-established air and water dispersion model to approximate the amount of released chemicals that would be likely to reach individuals, meaning that RSEI data include both air emissions (fugitive and stack) and water pollution, and only the incineration of off-site releases [11,19]. In turn, the RSEI model provides a Hazard Score, used to describe relative potential harm through the result of toxicity-weighted pounds, which take into account how much of a chemical is released into the environment, and how potent the chemicals are in causing health effects [19]. These score values are determined by multiplying a surrogate dose modeled by estimated environmental concentrations in pounds/year times a chemical and exposure route-specific toxicity weight [19]. Comprehensive information on the construction of the RSEI model and calculations used in its scores can be found in the EPA’s Risk-Screening Environmental Indicators (RSEI) Methodology Version 2.3.12.

RSEI scores, though, “are only meaningful when compared to each other”; scores are linearly related, meaning that “a computed RSEI Score value that is 10 times higher than another RSEI Score value suggests that the potential for risk-related impacts is 10 times higher” [2,19]. In Harris County in 2022, RSEI assigned risk values ranging from zero to over 231 billion for each chemical release from industrial facilities. For example, La Porte’s summative RSEI score for 1,2-dichloroethane was 9.42 × 10^9^, whereas Harrisburg/Manchester’s was 1.86 × 10^3^, suggesting that La Porte residents face a potential risk of developing adverse health effects from 1,2-dichloroethane exposure that is over 5 million times higher than that of Harrisburg/Manchester residents. In turn, RSEI scores do not indicate a specific threshold of chemical exposure deemed unsafe for civilians, but they do provide valuable insight by allowing communities to compare relative health risks and advocate for public health improvements [19]. Despite its limitations, the RSEI model remains the best way to predict the risk of industrial-related public health threats from industrial facilities in a given community.

To analyze chemical specific public health risks, data for environmentally released industrial chemicals in Harris County, Texas, in 2022 were obtained from the U.S. EPA RSEI [20]. Like the TRI dataset, one can navigate to the EPA’s RSEI website to download such data as spreadsheets, (www.epa.gov/rsei (accessed 12 February 2025)) by clicking Ways to Get RSEI Results, scrolling to EasyRSEI, and opening the dashboard. EasyRSEI Dashboard Version 2.3.12 is the most up-to-date EPA interface that allows the public to select different dimensions and/or metric options to create custom tables of various RSEI scores. To examine industrial chemical releases’ potential health risks in Harris County in 2022, a table was downloaded containing the submission year, TRI facility name, county, chemical, and RSEI modeled hazard score, which is the RSEI score most representative of the potential harm of a particular quantity of chemical exposure [19]. Once downloaded, the sheet was filtered by releases in 2022 and latitude/longitude coordinates for the TRI facilities were appended, as the location of facilities with RSEI scores are not included in the downloadable sheet. A pivot table was created in Excel with the facility names and latitude/longitude coordinates in rows, and the particular chemical releases’ RSEI modeled hazard score in columns. Next, RSEI scores for metals and their respective compounds were combined in the analysis; for example, the chemical cells “lead” and “lead and lead compounds” were combined to be one, “lead and lead compounds”. It must be noted that 419 out of the 2133 reported chemical releases from TRI facilities had RSEI scores “not modeled” and therefore had RSEI modeled hazard scores of zero, which are not exempt from the analysis.

In QGIS, the RSEI spreadsheet was added as a layer in the same project and saved, with the layer properties’ symbology of the data represented with a graduated point, where the larger the point, the higher the collective sum of RSEI modeled hazard scores for an industrial facility. Then, the following steps were taken: join attributes by location (summary) and join to features in the CTA layer where the features intersect by comparing to the RSEI modeled hazard score data, with sum being the summary to calculate. After adjusting the symbology of the resulting joined later to be graduated by the sum specific chemical RSEI modeled hazard scores, geospatial representations of the potential adverse health risks resulting from industrial air pollution in Harris County were produced.

## 3. Results

### 3.1. Step One: Analyzing Harris County for the Spatial Distribution of Industrial Pollution Burden

**Figure 1:**
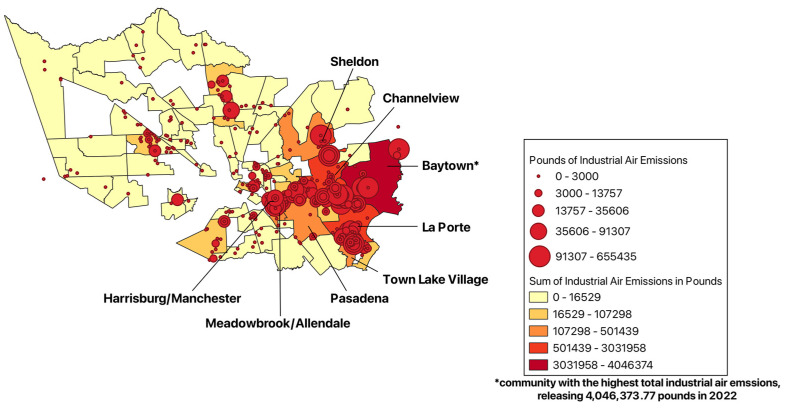
Total pounds of industrial air emissions in the communities of Harris County, 2022.

After evaluating the summative pounds of industrial air emissions within each CTA of Harris County, it was observed that the communities of Baytown, La Porte, Channelview, Harrisburg/Manchester, Meadowbrook/Allendale, Pasadena, Sheldon, and Five Corners are disproportionately affected by industrial air emissions (Figure 1). Note that the communities that did not have any industrial facilities to report any chemical release are blank.

**Figure 2:**
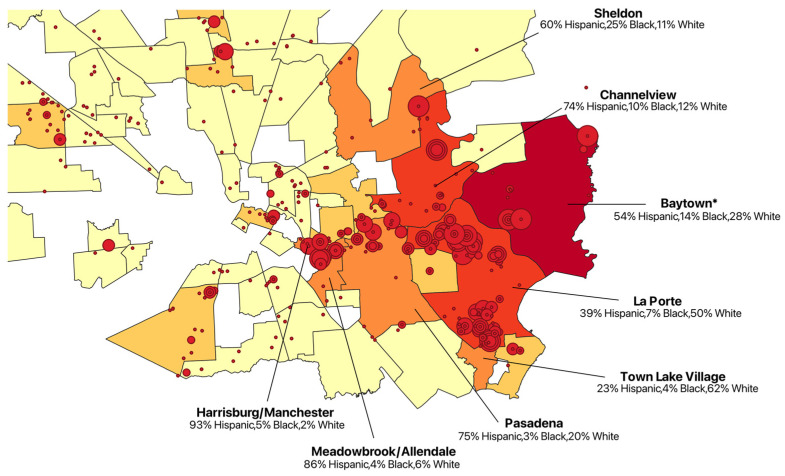
Reported ethnicities of Harris County communities facing the highest total pounds of industrial air emissions, 2022. * Baytown faced the highest total pounds of industrial emissions, releasing 4,046,373.77 pounds in 2022.

The Baytown community experienced the largest amount of industrial air emissions in 2022, summing to 4,046,373.77 lbs, followed by La Porte with 3,031,958.14 lbs, Channelview with 1,046,435.04 lbs, Pasadena with 501,439.13 lbs, Sheldon with 491,514.95 lbs, Meadowbrook/Allendale with 410,599.20 lbs, Harrisburg/Manchester with 409,181.74 lbs, and Town Lake Village with 336,540.05 lbs. After zooming in and examining these communities’ ethnic breakdown as of the Census’ ACS 2022 data collection, the following results were obtained (Figure 2).

**Figure 3:**
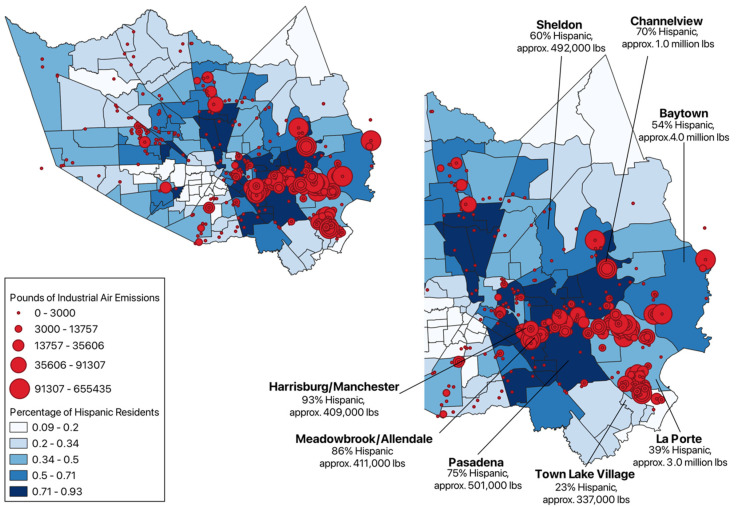
Percentage of Hispanic residents in Harris County communities in relation to industrial facility placement and total pounds of industrial air emissions, 2022.

Out of the eight most industrially polluted communities of Houston, six have a majority Hispanic population. To look into the demographics of communities in Harris County in relation to industrial facility pollution, the ACS—Five-Year Estimates—Frequently Used Variables dataset was used [15]. Once the ACS sheet was filtered by year (2022) and geo name (Community Tabulation Area) to produce the percentages of each ethnicity and race of CTAs, the data were imported to QGIS as a new layer for each demographic, with the layer properties’ symbology of the data graduated by each demographic percentage (Figure 3).

Based on these geographical representations, Hispanic communities appear to be disproportionately impacted by airborne industrial emissions. But these results are limited to indicating the source of airborne toxic releases, which does not necessarily equate to an increased risk of poor health outcomes. In turn, an assessment of individual chemical releases and their relative toxicity to nearby populations through RSEI modeled hazard scores is needed.

### 3.2. Step Two: Evaluating Individual Toxic Releases for the Spatial Distribution of Industrially Related Public Health Risk

Chemicals were chosen to have RSEI health risk by community visualizations and summations generated if they had substantial RSEI-modeled hazard scores relative to one another. The sixteen chemicals represented were ethylene oxide, propyleneimine, acetaldehyde, methyl iodide, 1,2-dichloroethane, chromium, cobalt, 1–3 butadiene, 1,1,2,2-tetrachloroethane, nickel, vinyl chloride, benzene, formaldehyde, arsenic, lead, and mercury. The results for the top seven most industrially polluted communities in Houston from Figure 2′s total RSEI-modeled hazard score for each of the chemicals are produced in Table 1.

The La Porte community has the highest summative RSEI-modeled hazard score, with an increased risk of negative health implications for all of the sixteen chemicals analyzed. The chemical companies with the highest RSEI-modeled hazard scores are Celanese (La Porte, TX, USA) (2.55 × 10^11^), Equistar Chemicals (La Porte, TX, USA) (1.09 × 10^11^), Dixie Chemical Co Inc. (La Porte, TX, USA) (8.34 × 10^10^), ExxonMobil (Baytown, TX, USA) (6.71 × 10^10^), LyondellBasell (Channelview, TX, USA) (5.66 × 10^10^), Albemarle Corp (La Porte and Pasadena, TX, USA) (2.54 × 10^10^), Oxy Vinyls LP (La Porte, TX, USA) (4.13 × 10^10^), and Eurecat US (La Porte, TX, USA) (1.28 × 10^10^). The most substantial RSEI-modeled hazard scores across the seven CTAs were for the chemicals 1,3-butadiene, benzene, and chromium.

**Figure 4:**
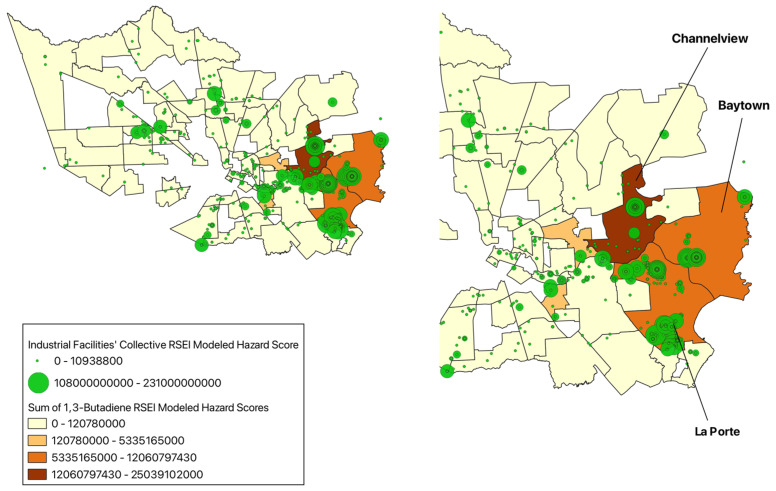
Sum of 1,3-Butadiene RSEI-modeled hazard scores from industrial facilities in the Harris County communities, 2022.

The communities with the highest RSEI-modeled hazard score for 1,3-butadiene are Channelview with a score of 2.50 × 10^10^, followed by Baytown with a score of 1.21 × 10^10^, and La Porte with a score of 8.10 × 10^9^ (Figure 4). 1,3-butadiene is used in the production of plastics, acrylics, and rubber, and is therefore emitted into the air from manufacturing and processing facilities [21]. This chemical is a classified human carcinogen, with studies showing associations between 1,3-butadiene exposure and increased incidence of leukemia [21]. At present, no information on reproductive or developmental effects of this chemical is available [21]. While there is robust evidence reflecting the hematological and carcinogenic effects of this chemical’s exposure, limited studies comparing the health effects of 1,3-butadiene between Hispanic and non-Hispanic populations exist. But there is evidence suggesting ethnic differences in the metabolism of 1,3-butadiene, with one study indicating that there could be ethnic variability in metabolic activation and detoxification pathways of the chemical [22]. In turn, further research is necessary to highlight potential differences in susceptibility or health outcomes related to Hispanic backgrounds.

**Figure 5:**
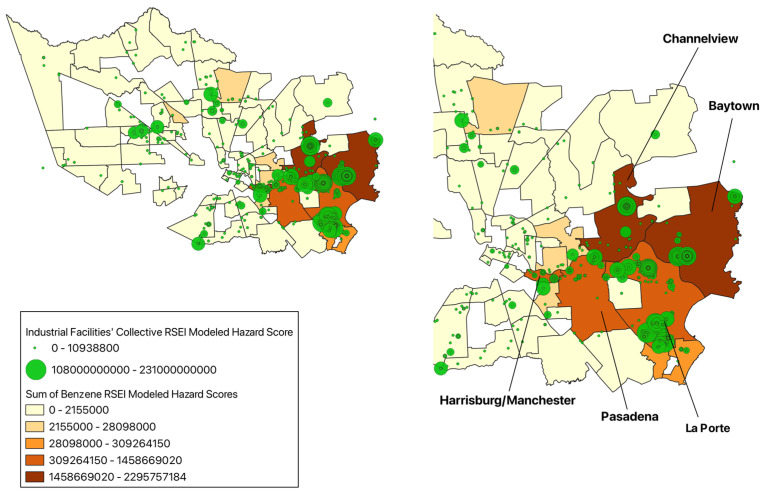
Sum of benzene RSEI-modeled hazard scores from industrial facilities in Harris County communities, 2022.

The communities with the highest RSEI-modeled hazard score for benzene are Channelview with a score of 2.30 × 10^9^, followed by Baytown with a score of 2.29 × 10^9^, La Porte with a score of 1.46 × 10^9^, Pasadena with a score of 9.58 × 10^8^, and Harrisburg/Manchester with a score of 8.61 × 10^8^ (Figure 5). Benzene is often found in the air due to the burning of coal and oil and the evaporation of industrial solvents, which can in turn be breathed in by individuals near the source of such emissions [21]. This chemical is a known human carcinogen from all sources of exposure (air and water), with long-term exposure causing blood disorders, reproductive effects in women, adverse effects on the developing fetus in animal tests, and increased incidences of leukemia [21]. From the existing literature, Hispanic populations appear to be disproportionately affected by benzene; one study found that cumulative cancer risks (CCRs) were statistically higher (*p* ≤ 0.05) in Hispanic individuals compared to non-Hispanic Whites, primarily due to increased Hispanic exposure to benzene [23].

**Figure 6:**
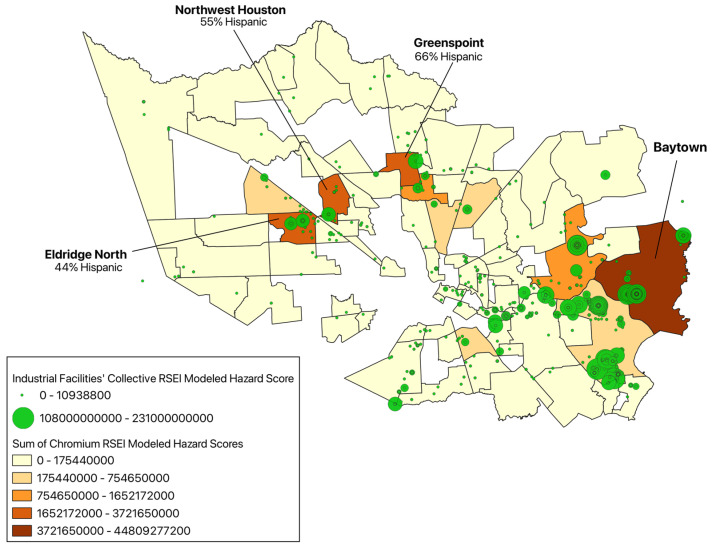
Sum of chromium RSEI-modeled hazard scores from industrial facilities in Harris County communities, 2022.

The communities with the highest RSEI-modeled hazard score for chromium are Baytown with a score of 4.48 × 10^10^, followed by Eldridge North with a score of 3.72 × 10^9^, Greenspoint with a score of 3.32 × 10^9^, and Northwest Houston with a score of 2.41 × 10^9^ (Figure 6). Chromium is used industrially mainly for making steel and other alloys. Chromium (VI) compounds, more often than chromium (III) compounds, are released into the environment due to ferrochrome (stainless steel) production, ore refining, chemical and refractory processing, and cement-producing plants [21]. Chromium (VI) is much more toxic than chromium (III), but both compounds target the respiratory tract in humans—although the body can detoxify some amount of (VI) to (III) [21]. Chromium (VI) is a clearly established human carcinogen, with chronic exposure primarily associated with an increased risk of lung cancer, along with immune system alterations and kidney disease [21]. There is a limited understanding of how chromium (VI) may complicate pregnancy, childbirth, and development [21]. Although it is evident that people who live near industrial plants are more likely to experience chromium exposures, which are generally mixed forms of chromium (VI) and chromium (III), the existing literature does not specially address differences in health effects between Hispanic and non-Hispanic populations [21]. It must be noted that the RSEI model considers toxicity values for the more toxic chromium (VI) compound [17].

To conclude, Latinx communities are at a disproportionate, increased risk of adverse health effects from industrial facilities according to summative geospatial analysis of 2022 data.

## 4. Discussion

Access to use and interpret industrial environmental health data and demographic information can have several positive policy implications for state, regional, and national environmental offices [24]. GIS analysis of industrial releases and health risks using RSEI data, along with demographic information, pays particular attention to underrepresented communities that could be used by regional EPA offices to ensure that risk reductions will be made in these areas [24]. Additionally, state agencies nationwide could use these data to create state-level initiatives to encourage high-polluting and high-risk score facilities to reduce emissions or substitute their production processes for a less toxic alternative [24,25].

This geospatial analysis of TRI and RSEI data revealed that in 2022, six of the eight Harris County communities most burdened by industrial emissions were predominately Latinx: Sheldon, Channelview, Baytown, Pasadena, Meadowbrook/Allendale, and Harrisburg/Manchester. These findings also highlighted the resulting disproportionately increased risk of adverse health effects, particularly from 1,3-butadiene, benzene, and chromium, among a multitude of chemicals. By describing the process of uncovering who is most exposed to health-threatening industrial pollution in Houston, this methodology paper highlights (1) a violation of environmental justice in Latinx communities of Houston, and (2) how the EPA’s public data resources may not be useful for impacted communities to analyze in advocating for improvements in public health. The process has shown that many resources are required to generate data-backed evidence of industrially related health risks from publicly available EPA resources: it is necessary to have (1) appropriate education in chemistry, statistics, and math, (2) access to the internet, along with spreadsheet and geographical information software, (3) resources for consultation, and (4) adequate time. These resources are often something that underserved Latinx communities may not have, which increases the difficulty of analyzing, interpreting, and providing evidence for proof of disproportionate industrial exposures and increased risk of negative health outcomes.

Without experts who specialize in environmental exposure research, community members must sacrifice other important aspects of their work to take the extended time needed for a person without the required expertise to complete a challenging data analysis [19]. Additionally, environmental justice organizations see GIS as a powerful visualization tool that can be used to match RSEI data with demographic information, being capable of influencing the minds of individuals in power, but recognize it as software that requires specific technical expertise that community members often do not have access to [19].

This analysis reflects that the Latinx communities of Houston are not experiencing the “fair treatment” defined by the EPA as bearers of disproportionate industrial pollution and increased risk of associated health problems. As shown from the described processes to access, analyze, and interpret TRI and RSEI data, using EPA resources can be especially difficult for lower-resourced individuals who are often the ones who experience overwhelming industrial pollution. This was reflected in the TRI’s initial years, when higher-educated communities were able to overcome the database’s obstacles to aid in reducing emissions in their neighborhoods, suggesting that the EPA’s resources do not benefit all groups of people equally, let alone the groups that need it the most, such as Latinx communities of Houston.

Community-based participatory research (CBPR) on industry activities has already led to public health improvements in Houston, reflecting that greater accessibility to EPA resources could empower communities to conduct data-driven analyses and advocate for public health reforms [26]. A notable example is the Metal Air Pollution Partnership Solutions (MAPPS) initiative, which brought together academia, the environmental justice organization Air Alliance Houston, the metal recycling industry, the local health department, and community members [26]. Through community collaborative data collection and analysis, this initiative helped develop a comprehensive public health action plan aimed to reduce metal aerosol emissions and related health effects, an approach that could also be applied to TRI facilities [26].

## 5. Limitations

Despite the advanced technology of GIS software, one must note the important limitations of such analysis techniques in an environmental injustice and public health context. The most significant shortcoming of using a software like QGIS to examine community pollution burden is its failure to recognize boundary or edge effects; GIS software assumes that pollution is solely confined to its spatial unit [12,27]. When using a GIS software, if one neighborhood has a polluting source within their spatial boundaries, it is considered impacted by it, while if another neighborhood does not have a polluting source within it, it is not considered to be impacted [12,27]. This could cause issues if, for instance, an individual lives within the same community as a polluting facility yet far away from it and still is considered to be facing exposure consequences, while another individual living right across the street from a polluting facility, but because their home is not technically within that community, they are not considered affected by it [12,27]. Another limitation of GIS in an environmental health injustice context is that such software requires access to more advanced technical expertise and skills that many under-resourced communities may lack [13].

Additionally, the Census’ ACS data are flawed due to their potential undercounting of Latinx individuals. All people with a residence in the United States, regardless of age, citizenship, or immigration status, are included in the population for the Census [28]. Naturally, ethnoracial classification systems are different in different countries, so immigrants are often less familiar with the United States classifications of ethnicity such as “Hispanic or Latino origin”—the verbiage used in the Census [28]. Some suggest that the terms “Hispanic” or “Latino” are solely a creation used by the United States and are therefore meaningless in Latin America, potentially leading to an incorrect response by an individual and a missing person in the official population count [28]. Moreover, many Latinx individuals do not identify with the category “Hispanic”, causing more to opt out of this Hispanic origin question and be missed in the official population count [29]. These reasons lead many to believe that there is a higher probability that the Hispanic community has experienced an ethnic undercount in the Census [29]. This undercounting of Latinx individuals due to a classification error may make it appear as though the size and magnitude of social injustices facing this community, such as poverty, economic opportunity, fair housing, and more, are less than reality [29]. Without a correct understanding of the Latinx population and the scope of the problems they face, misaligned and ineffective policies are formed, which does not help this population [29].

Likewise, there are many limitations to the EPA’s TRI and RSEI resources. Despite the extensivity of the TRI database, the information conveyed in the Toxics Tracker and the datasets is not completely representative of the environmental pollution burden and associated health risks. To begin, the only industrial facilities required to report annual chemical releases are the ones that qualify via the criteria mentioned prior, which means that facilities just below the reporting thresholds, who may be contributing to overall air emissions just as much or more, are not required to report to the EPA’s TRI [12,18]. Additionally, one of the major contributors to air pollution is method of transportation, which is not included in TRI data and therefore not accounted for [12]. This is typical of even the best of state and federally compiled environmental data, which often have huge information gaps [12].

The greatest limitation of RSEI scores in providing industrial public health risk is that they “are only meaningful when compared to each other” due to their linear relationship with one another and lack of a scale [2,19]. This means that although RSEI may be useful in providing risk context for industrial emissions, it is not a risk assessment that quantifies the effects of chemical exposure in populations or single individuals; for example, a risk assessment could find that “a carcinogen in drinking water is associated with a 10-fold elevation in cancer risk” [2]. Although this would be useful information for the public to have and use in public health and environmental injustice circumstances, the EPA fails to incorporate risk assessments in TRI and RSEI databases [2].

While discussing the limitations of RSEI, it must be mentioned that there have been many simplifying assumptions made in the creation of the model, like how each facility in the database is given one smokestack height estimate that is often based on the median smokestack height for the whole industry [27]. These modeled estimations may be of concern, but some information that should be of more importance is the fact that many chemical releases from TRI facilities are not modeled by RSEI at all. For instance, if one downloads the Excel TRI and RSEI spreadsheets for Harris County in 2022, one will find that TRI logged 2164 chemical releases from industrial facilities, RSEI logged 2133 chemical releases, and 419 of those RSEI chemical releases had scores “not modeled”. Consequently, the RSEI data reflected in the more easily understandable TRI Toxics Tracker (Figure 3) are not entirely representative of all TRI facility releases, let alone all chemical exposures.

Regardless of their shortcomings, TRI and RSEI data are the U.S.’s gold standard industrial chemical release database, and TRI has been influential in shaping public policy through pollution prevention, even if access to such data may influence reduced emissions in predominately White, higher-income communities [24].

## 6. Recommendations

Having public resources such as TRI and RSEI available on the internet as a sole means of disseminating industrial-related public health information is biased towards higher-income and more-educated groups [24]. At the same time, EPA resources offer “open” access to documents that require technical knowledge to interpret, making them inaccessible to non-expert, often under resourced, communities [30]. A way to address these discrepancies could involve the EPA applying its own definitions to implement changes in their public resources so that they can benefit all. With the ability to use and interpret EPA data, communities that need to provide evidence for environmental justice can influence policy changes for better living conditions. First, state agencies should adopt information dissemination policies that inform communities of the EPA resources’ existence through outreach venues and public meetings [24]. Primarily, though, to make EPA resources more utilizable for Latinx communities, it is necessary for the Agency themselves along with state agencies to provide additional assistance for such individuals to use and interpret emissions-based data such as TRI and RSEI [24]. This way, the data required to provide proof of environmental injustice can be more easily obtainable and interpretable to benefit the communities that need them the most. One way this could be possible is to create employment positions filled by individuals who speak both English and Spanish with post-graduate education in environmental and public health. By providing their contact information on the EPA’s website, community members interested in receiving help cleaning and/or analyzing data can have a resource to reach out to.

As mentioned previously, various forms of GIS software like QGIS are extremely compelling forms of data presentation due to their visualization components, but this software is not something that an individual can learn on their own with the help of YouTube videos and Google searches. To alleviate this difficulty, software companies could have expert, bilingual employees host lessons in historically underrepresented or lower-income schools and community centers to teach these individuals how to take spreadsheet data and analyze them on platforms like QGIS. This way, the lack of resources to learn to use effective software would be less of a barrier for communities that could use these results the most.

Likewise, it is necessary to better account and represent Latinx individuals within Census data to fully understand the scope of such environmental justice issues [29]. Improving the accuracy of Latinx demographic data in the Census may be achieved by building trust and increasing participation within these neighborhoods through the hiring of census workers from within the communities themselves, or from CBPR to collect population data.

## 7. Conclusions

By providing the steps required to map public industrial emissions and health risk data using QGIS, this paper aims highlight the disproportionate burden of industrial pollution in Houston’s Latinx communities and the associated increased risk of adverse health effects from exposure to a multitude of chemicals, particularly 1,3-butadiene, benzene, and chromium. Additionally, this study uncovers the reality of how inaccessible analysis and interpretation of EPA datasets can be for under-resourced communities. As a result, Latinx communities, such as those in Houston disproportionately affected by industrial pollution, may be less likely to utilize public EPA resources or online software to provide scientifically supported proof of such public health risks, further perpetuating the cycle of environmental injustice. While the methodology described serves as a useful guideline for identifying communities experiencing environmental injustice, greater efforts from the EPA and GIS software companies are needed to enhance accessibility. This could include initiatives such as regional EPA data assistance personnel or GIS community outreach programs to provide hands-on training and support.

## Figures and Tables

**Table 1:** Total RSEI-modeled hazard score per chemical by Harris County community, 2022.

Chemical	Summative RSEI Modeled Hazard Score by CTA *						
	La Porte	Baytown	Channelview	Pasadena	Meadowbrook/ Allendale	Harrisburg/ Manchester	Sheldon
All chemicals (total RSEI modeled hazard score)	5.50 × 10^11^	7.23 × 10^10^	5.70 × 10^10^	7.86 × 10^9^	5.39 × 10^9^	2.61 × 10^9^	1.33 × 10^7^
Ethylene oxide	3.55 × 10^11^	0	2.65 × 10^10^	0	0	0	0
Propyleneimine	4.76 × 10^5^	5.81 × 10^5^	1.15 × 10^5^	2.43 × 10^4^		2.46 × 10^4^	0
Acetaldehyde	1.18 × 10^10^	0	2.32 × 10^8^	1.06 × 10^7^	0	5.60 × 10^5^	0
Methyl iodide	1.02 × 10^10^	0	0	0	0	0	0
1,2-dichloroethane	9.42 × 10^9^	0	0	0	0	1.86 × 10^3^	0
Chromium	7.49 × 10^8^	4.48 × 10^10^	1.65 × 10^9^	2.47 × 10^5^	1.08 × 10^7^	0	3.12 × 10^6^
Cobalt	3.21 × 10^10^	1.18 × 10^9^	0	0	0	0	0
1,3-butadiene	8.10 × 10^9^	1.21 × 10^10^	2.50 × 10^10^	1.21 × 10^8^	5.34 × 10^9^	9.91 × 10^7^	0
1,1,2,2- tetrachloroethane	1.37 × 10^10^	0	0	0	0	0	0
Nickel	1.79 × 10^9^	6.92 × 10^9^	2.43 × 10^4^	8.89 × 10^7^	0	2.54 × 10^4^	1.18 × 10^6^
Vinyl chloride	5.56 × 10^9^	0	0	0	0	0	0
Benzene	1.46 × 10^9^	2.29 × 10^9^	2.30 × 10^9^	9.58 × 10^8^	2.81 × 10^7^	8.61 × 10^8^	0
Formaldehyde	8.26 × 10^8^	2.39 × 10^9^	0	1.25 × 10^7^	0	1.93 × 10^3^	0
Arsenic	1.04 × 10^8^	0	0	0	0	0	0
Lead	1.11 × 10^7^	1.66 × 10^7^	5.10 × 10^5^	5.56 × 10^5^	0	6.58 × 10^5^	8.34 × 10^6^
Mercury	1.39 × 10^6^	4.09 × 10^6^	9.84 × 10^3^	1.19 × 10^4^	0	8.40 × 10^4^	0

* RSEI-modeled hazard scores are linearly related. For example, La Porte’s summative RSEI score for 1,2-dichloroethane is 9.42 × 10^9^, whereas Harrisburg/Manchester’s is 1.86 × 10^3^. This suggests that La Porte residents face a potential risk of developing adverse health effects from 1,2-dichloroethane exposure that is over 5 million times higher than that of Harrisburg/Manchester residents.

## Data Availability

The shapefile for Harris County CTAs in 2020 used in the analysis is openly available at https://www.kinderudp.org/#/datasetCatalog/6ep1wpn43red (accessed on 5 June 2024). Census’ ACS—Five-Year Estimates 2022 data used in the analysis are openly available at https://www.kinderudp.org/#/datasetCatalog/19z57m7ak5ov (accessed on 5 June 2024). TRI data for Texas in 2022 used in the study are openly available at https://www.epa.gov/toxics-release-inventory-tri-program/tri-basic-data-files-calendar-years-1987-present (accessed on 5 June 2024). RSEI data for Texas in 2022 used in the study are openly available at https://edap.epa.gov/public/extensions/EasyRSEI/EasyRSEI.html# (accessed on 5 June 2024).
